# Analysis of Bacterial and Fungal Communities and Organic Acid Content in New Zealand Lambic-Style Beers: A Climatic and Global Perspective †

**DOI:** 10.3390/microorganisms13020224

**Published:** 2025-01-21

**Authors:** Aghogho Ohwofasa, Manpreet Dhami, Christopher Winefield, Stephen L. W. On

**Affiliations:** 1 Department of Wine, Food and Molecular Biosciences, Lincoln University, Lincoln 7647, New Zealand; aghogho.ohwofasa@lincoln.ac.nz (A.O.); christopher.winefield@lincoln.ac.nz (C.W.); 2 Centre of Foods for Future Consumers, Lincoln University, Lincoln 7647, New Zealand; 3 Manaaki Whenua—Landcare Research, Lincoln 7640, New Zealand; dhamim@landcareresearch.co.nz

**Keywords:** lambic beer, spontaneous fermentation, microbial diversity, metabarcoding, climate change

## Abstract

Beer produced by autochthonous microbial fermentation is a long-established craft beer style in Belgium that has now been implemented commercially in New Zealand. We used a metabarcoding approach to characterize the microbiome of 11 spontaneously fermented beers produced by a single brewery in Oamaru from 2016 to 2022. Key organic acid concentrations were also determined. Both bacterial and fungal populations varied considerably between vintages and between individual brews produced in 2020. Similarly, for organic acids, the concentrations of L-malic acid, succinic acid, and L-lactic acid statistically differed from one vintage to another. Moreover, a correlation between the concentrations of certain organic acids and microbial composition was inferred by ordination analyses. Through reference to publicly available climate data, humidity and maximum temperature seemed to enhance the abundance of *Penicillium* and *Hanseniaspora* in beer microbiota. However, comparison with previously published studies of Belgian lambic beers, similar Russian ales, and publicly available temperature data from these regions showed that the microbial populations of these were relatively stable despite greater extremes of weather. Our results suggest that while climatic variables may influence microbial populations during beer making that employs autochthonous fermentation in New Zealand, such variation is not evident where similar beers are produced in facilities with a long-established history of production. These findings have implications for lambic-style beer production in the context of global climate change, notably where microbial populations may lack environmental adaptation.

## 1. Introduction

Beer is a widely consumed beverage on a global scale [1]. To achieve consistency, it is common for beer industries to use yeast starter cultures in producing beers [2,3]. However, a spontaneous fermentation process can be used; these beers are usually left to mature in wooden barrels for a period of one to three years [4]. The earliest known (13th century) beverage of this style is called lambic beer, which is produced in the Pajottenland region, surrounding the valley of the Zenne River in the southwestern part of Brussels, Belgium [5,6]. Similar to the “*terroir*” concept associated with wines, it is believed that the Zenne valley contains important microorganisms in the open air that are necessary for the fermentation and maturation of lambic beer wort [6]. Due to these specific environmental characteristics, some authors argue that beers made outside this region do not meet the requirements of the native product [7]. Nonetheless, other mixed fermentation beers produced spontaneously that mimic the lambic beer style are popular [2]. For example, American Coolship Ales (ACAs) are made in regular breweries as a seasonal product in the USA [4]; the microbiota associated with these products is said to resemble lambic beers [8]. Inevitably, the use of autochtonous microflora to drive the fermentation process results in a diverse microbial population that includes various yeast species, lactic acid- and acetic acid-producing bacteria, ultimately resulting in beers that are biochemically and organoleptically complex due to the presence of ethanol, organic acids, and many other products also [6].

In New Zealand, the beer industry has witnessed substantive growth and was reported to have contributed NZD 3.3 billion to the New Zealand economy in the year 2022 [9]. In terms of consumption, 294 million liters were consumed in the year 2022—the most for alcoholic beverages in the country [10]. However, few New Zealand brewers employ spontaneous fermentation processes for production.

Globally, few studies have assessed the microbiota associated with the fermentation and maturation of beer as well as finished beer products. More recently, there has been an increase in the use of targeted sequencing (amplicon-based sequencing, commonly known as metabarcoding) and untargeted amplification (metagenomics) in understanding convoluted microbial communities. This is because of the advantages it offers in investigating the unculturable world of microbes [11]. They have been used to determine the fungal and bacterial microbiota associated with wine and musts fermentations [12,13,14], but few studies have examined beers produced using spontaneous fermentation methods. The “BeerDecoded” project used Internal Transcribed Spacer metabarcoding to examine 39 bottled beers from five countries, and it determined a low abundance but an extensive diversity of fungal species [15]. Bacterial species were not sought for, and the number of beers produced by spontaneous fermentation was not declared in this study. Elsewhere, studies have only examined such beers from Russia and Belgium [1,2,16]. New Zealand beers have not previously been studied.

Oamaru is a traditional beer brewing town in the South Island of New Zealand that has been gaining in popularity by craft brewers in recent years. The climate in this region is categorized as warm and temperate. It appears unique in that “there is a considerable amount of rainfall even during months that typically experience dry weather” [17]. In this study, we apply a metabarcoding approach to determine the bacterial and fungal microbiomes of beers produced in the Oamaru region of New Zealand over six vintages. We compare our results to those of previously published studies of Belgian and Russian beers [1,2,16], and we explore the role that climatic variables may play in the variation observed. We also determine the concentrations of each of five organic acids in these samples to evaluate any correlations between microbial populations and biochemical profile.

## 2. Materials and Methods

### 2.1. Beer Production and Sampling

Beer used in this study was made by a single, boutique craft brewery located in Oamaru, New Zealand (45.0966° S, 170.9714° E) using a spontaneous approach similar to Belgian lambic beer [6]. The brewer utilized malted pilsner and unmalted wheat with 300 g of hops dried and aged for one year added to form the wort. The wort was boiled for 3 h and transferred into a large cooling vat, which was thoroughly cleaned beforehand. Thereafter, the wort was left outside overnight. After cooling to a temperature of 20 °C, the sugar concentration was established to be 13 °P (13 g of sugar per 100 g of wort). The cooled wort (without filtration) was transferred to large casks of 220 L where fermentation and post-fermentation brewing occur for 1 year. The casks used were retired barrels formerly used in the aging of Pinot noir wines and were flushed with water prior to use. End point (i.e., fermentation complete) samples of 100 mL were collected aseptically using the valve in front of the barrels. The average temperature of the cellar ranged from 15 °C to 20 °C degrees throughout the year. End point beer samples from several vintages and years from the brewery were collected and examined. Supplementary Table S1 details sampling information. All samples collected were stored at −80 °C prior to processing.

### 2.2. DNA Extraction and Sequencing

We utilized the approach employed by Tyakht et al. [1] to generate pellets from the beer samples, centrifuging 50 mL of each sample at 4700× *g* for 10 min at 4 °C (Heraeus Multifuge X3R, Thermo Scientific, Waltham, MA, USA). The pellet generated was kept at −20 °C for DNA extraction.

The Mag-Bind Environmental DNA 96 kit (Omega, Norcross, GA, USA) was used for DNA extraction, as per the manufacturer’s recommended protocol with slight adjustment. We modified the cell disruption step where we employed a TissueLyser II (Qiagen, Venlo, The Netherlands) for 3 min. A 1.5% agarose gel electrophoresis was used to estimate the quality of all extracted DNA. We then measured the total DNA concentration using a DeNovix DS-11 spectrophotometer (Wilmington, DE, USA).

For metabarcoding, we applied the two-step amplification process used by Ohwofasa et al. [14]. The first step involves PCR amplification of the large subunit (LSU) ribosomal RNA gene region using an equimolar mix of the primer pairs LSU200(A)-F/LSU481(A)-R and LSU200-F/LSU481-R for fungal community analysis (Forward 200AF: NNNNNNAACKGCGAGTGAAGCRGYA; 200F: NNNNNNAACKGCGAGTGAAGMGGGA Reverse 481R: NNNNNNTCTTTCCCTCACGTTACTC; 481A-R: NNNNNNCSATCACTSTACTTGTKCGC) [18]. The V4 region of the 16S ribosomal RNA gene targeted using the 515F/806R primers (Forward: GTGCCAGCMGCCGCGGTAA; Reverse: GGACTACHVGGGTWTCTAAT) was used for bacteria community analysis [19]. PCR for the first step was carried out using a KAPA 3G PCR plant kit (Sigma-Aldrich, St. Louis, MO, USA). The PCR conditions were as follows: initial denaturation at 95 °C for 120 s, followed by 35 cycles at 95 °C for 20 s (denaturation), 55 °C (fungal)/52.5 °C (bacterial) for 20 s (annealing), 72 °C for 30 s (extension), and a final extension at 72 °C for 10 min.

The PCR products of the first step PCR served as template DNA for the second PCR. Barcoded primers were used for amplification with the following PCR conditions: initial denaturation at 95 °C for 2 min, 5 cycles of 95 °C for 20 s (denaturation), 50 °C for 20 s (annealing), 72 °C for 30 s (extension), and final extension at 72 °C for 2 min. The resulting PCR products were cleaned up using SeraMag Magnetic Speed-Beads (Merck, Darmstadt, Germany) [20] to remove primer dimers and normalize sample concentration. We quantified these samples using Qubit (dsDNA HS Assay Kit, Invitrogen, Carlsbad, CA, USA). Upon quantification, we pooled samples in an equimolar manner accounting for the number of samples and amplicon length each library contains. The quality of the final pooled library was evaluated using a LabChip GX Touch Nucleic Acid Analyzer (PerkinElmer, Waltham, MA, USA). Sequencing was performed by the Auckland Genomics Facility (University of Auckland) utilizing the Illumina MiSeq platform (phiX spike 10%, 250 × 2 cycles, NanoSeq kit) (Illumina, San Diego, CA, USA).

### 2.3. Amplicon Sequence Variant (ASV) Cluster and Annotation of Species

With the aid of bcl2fastq2 (version 2.20) conversion software, raw data in Bcl file formats were converted to fastq format. Demultiplexing was performed using Claident (version 2018.05.08) [21]. The amplicon-based DADA2 (version 1.20.0) pipeline [22] was used for quality filtering, merging, chimera removal, and inferring of amplicon sequence variants (ASVs). Eukaryotic taxonomic assignment was performed using the UNITE fungal taxonomic reference [23]. For prokaryotes, we used the SILVA v132 16S rRNA database [24]. Occasionally and where applicable, we probed ASVs against the Basic Local Alignment Search Tool (BLAST) [25] to achieve a better taxonomic resolution. Bioinformatics analysis was carried out using Jupyter via the New Zealand eScience Infrastructure (NeSI) HPC environment.

### 2.4. Climate Data

The weather reports associated with all vintages were accessed from the Ostler Vineyard Weather Station [26]. This station is situated in the north of Otago close to the location of the brewery. Monthly data were collected (Supplementary Table S2), averaged, and used subsequently as our climate data.

The Belgian lambic beers for which microbiome data have been determined were brewed in an area southwest of Brussels [2,16]. We purchased daily temperature data for Brussels for the vintages examined (2014–2017) by these authors from Weatherspark (https://weatherspark.com/) to establish ranges and means for comparison.

### 2.5. Organic Acid Determination Using HPLC

Using High-Performance Liquid Chromatography (HPLC) (Shimadzu Corporation, Kyoto, Japan), the method utilized by Shi et al. [27] was employed. In addition to acetic acid, which was sourced from BDH, all chemicals used were purchased from Sigma-Aldrich (Sigma-Aldrich, St. Louis, MO, USA). Separation and analyses of organic acids was achieved using the Rezex ROA-Organic Acid H+ (8%) column (3000 × 7.8 mm, Phenomenex, Torrance, CA, USA) which had a Guard column (Carbo-H 4x3.0; Phenomenex). The mobile phase (5 mM H_3_SO_4_) was filtered through a 0.45 µm membrane. The column temperature was fixed at 55 °C with a flow rate of 0.5 mL/min. A sample volume of 20 µL was injected, and the UV detector (SPD-20A, Shimadzu, Kyoto, Japan) was set at a wavelength of 210 nm. A mixture of the standard stock solution was prepared using analytical grade formic acid, D-gluconic acid, acetic acid, citric acid, L-malic acid, oxalic acid, succinic acid, and L-lactic acid. The standard stock solution was then used to prepare standard curve dilutions. The standard curve concentrations were 0, 1.0, 2.0, 5.0, 10.0, 20.0, 50.0, 100.0, 300.0, 500.0, 1000.0 ppm, respectively, in 5 mM H_2_SO_4_. Sample preparation was achieved by adding 1.8 mL 5 mM H_2_SO_4_ to a 0.2 mL beer sample. After mixing properly, this was filtered through a 0.2 µm PVDF membrane filter. Filtered samples were further diluted 10 times with 5 mM H_2_SO_4_ prior to injection. After comparing the retention time of organic acid standards, the identification of all organic acids present in the beer samples was possible. Sample quantification was performed using the peak area of the chromatograms from the external calibration standard curve. All data were processed using the Lab solution software (Version 5.87 SP1).

### 2.6. Statistical Analysis

We carried out statistical analysis using the open-source R programming language [28] (v4.1.0). The Phyloseq package (v1.38.0) [29] was used to create one fungal and bacterial phyloseq object. Several other packages were also utilized (see Supplementary File S1). The core microbiome (ASVs detected in 70% of the samples with an abundance ≥ 0.0001) associated with beer was identified after transforming ASV abundances to their relative abundance. This was necessary to account for dissimilarities in library sizes. Alpha diversity (Shannon diversity index) was calculated to determine differences within each sample. The function Betadisper in the R package “vegan” was applied for estimating dispersion distances between communities. The distances were visualized using Principal Coordinates Analysis (PCoA). We employed the standard parametric ANOVA and permutation tests for homogeneity (permutest) to scrutinize the statistical significance of the betadisper object.

To determine ASVs that were significantly different, DESeq2 was used [30]. Depending on the results of the first axis of the decoruna function in vegan [31,32], we utilized either redundancy analysis (RDA) or canonical correspondence analysis (CCA) to visualize any association between the climatic data associated with a given vintage and its microbiome. The same approach was used to uncover any correlation between microbial abundance and organic acid concentrations. The model that optimizes the variation explained while using fewer variables was selected using a forward selection approach. When RDA was utilized, we transformed our data using Hellinger’s transformation.

## 3. Results

### 3.1. Climatic Variables in Oamaru, New Zealand

A total of seven climatic variables in all vintages from Oamaru were assessed, and these are shown in Table 1.

### 3.2. Temperature Ranges in Brewing Locations of New Zealand and Belgium

Table 2 displays the temperature minima, maxima and mean for the Oamaru and Brussels locations across the relevant beer production periods studied here (Oamaru) and in Belgium (2014–2017; cf.) [2,16].

### 3.3. Bacterial and Fungal Community Composition in All Vintages

**Figure 1:**
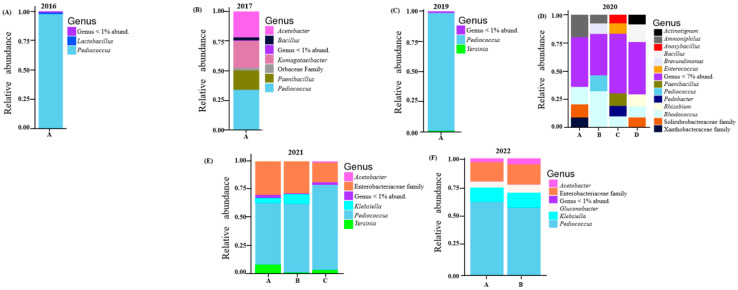
Relative abundance of the core bacterial genera in vintage: (**A**) 2016; (**B**) 2017; (**C**) 2019; (**D**) 2020; (**E**) 2021; (**F**) 2022. Some bacteria which were not resolved with certainty to the genus level are shown at the family level. Vintages with multiple barrels are indicated with different letters.

The relative abundance and richness of the core bacterial communities detected in all vintages are shown in Figure 1. In five of the six vintages examined, *Pediococcus* spp. were detected in significant proportions; only in the 2020 vintage, which had a notably higher richness and diversity (Figure 1D), was this trend not evident. In terms of specific composition, upon taxonomic assignment, the 229 amplicon sequence variants (ASVs) gave rise to a total of 78 bacterial genera. The 2016 vintage was completely dominated by *Pediococcus* (97.6%); other genera detected included *Lactobacillus* (1.24%), *Komagataeibacter* (0.18) and *Bacillus* (0.13%). However, in the 2017 vintage, *Pediococcus* was not as predominant, as seen in the 2016 vintage. Here, *Pediococcus* made up 33.9%. *Komagataeibacter* (22.7%), *Acetobacter* (21.7%), and *Paenibacillus* (16.3%) were the other common genera associated with this vintage. The 2019 vintage was similar to the 2016 vintage where *Pediococcus* showed high relative abundance at 97.5%; however, *Rhodococcus* (0.08%) and *Acetobacter* (0.33%), which were not detected in previous vintages, were present here in low relative abundance. All four barrels sampled in the 2020 vintage indicated that this vintage was different in terms of bacterial composition. *Pediococcus* was visibly detected in only one of the four barrels, and it was in a far lower proportion (ca. 15%) than observed in all other vintages. *Rhodococcus* thrived in this vintage, as it was detected in all four barrels, as high as 32% in barrel B. The 2021 vintage was again dominated by *Pediococcus*, *Klebsiella* and members of the Enterobacteriaceae family. Together they made up over 85% of bacteria genera detected in all three barrels of this vintage. The same trend was observed in the 2022 vintage. *Acetobacter*, *Ammoniphilus* and *Gluconobacter* were also present but in low proportions (Figure 1 and Supplementary Table S3).

**Figure 2:**
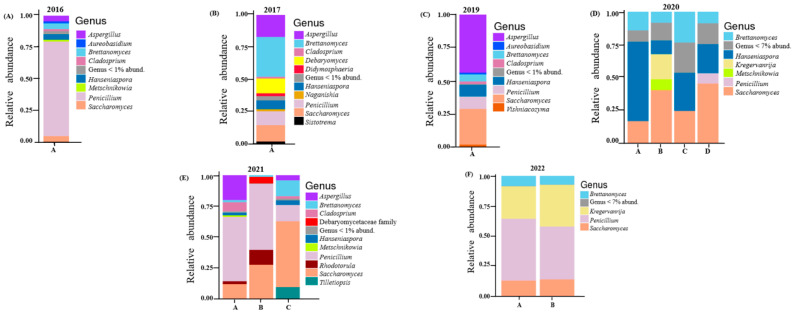
Relative abundance of the core fungal genera in vintage: (**A**) 2016; (**B**) 2017; (**C**) 2019; (**D**) 2020; (**E**) 2021; (**F**) 2022. Fungi which were not resolved to the genus level are shown at the family level. Vintages with multiple barrels are indicated with different letters.

A total of 107 fungal ASVs resulted in 27 fungal genera after taxonomic assignment. The relative abundances of these are shown in Figure 2 below. The 2016 vintage had a large proportion of *Penicillium* (74.8%). Others detected includes *Saccharomyces* (4.67%), *Aspergillus* (4.55%), *Hanseniaspora* (4.33%) and *Brettanomyces* (4.5%). *Cladosporium, Rhodotorula* and *Torulaspora* were found in small amounts (all making up less than 2%). The most abundant fungi in the 2017 vintage were *Brettanomyces* (31%). This was followed by *Aspergillus* (17.1%), *Saccharomyces* (12.8%), *Debaryomyces* (11.4%) and *Penicillium* (10.7%). Genera unique to this vintage include *Naganishia* (1.7%) and *Pichia* (0.16%). *Aspergillus*, the filamentous fungi, was seen to be dominant (44%) in the 2019 vintage. *Saccharomyces* (26.9%), *Penicillium* (9.34%), *Hanseniaspora* (8.8%) *Brettanomyces* (5.4%) and *Cladosporium* (1.9%) were also reported.

All four barrels of the 2020 vintage were identical with *Saccharomyces*, *Hanseniaspora* and *Brettanomyces* constituting more than 60%. In this vintage, barrel B was unique with the presence of *Kregervanrija* (19%) and *Cystofilobasidium* (4.29%). Two barrels (A and B) of the 2021 vintage had high amounts of *Penicillium* (over 50%). Other genera detected in barrel A were *Aspergillus* (19.1%), *Saccharomyces* (12%), *Cladosporium* (7.2%), *Rhodotorula* (2.4%) and *Brettanomyces* (1.9%). Barrel B, on the other hand, had a higher proportion of *Saccharomyces* (27.4%) and *Rhodotorula* (12%) when compared to barrel A. The genus *Debaryomyces* (5.2%) was only detected in barrel B of this vintage. *Saccharomyces* (53%) was the highest detected in barrel C. Others included *Penicillium* (13%), *Brettanomyces* (12.8%) and *Tilletiopsis* (10%)*. Penicillium, Saccharomyces, Kregervanrija* and *Brettanomyces* were dominant in the 2022 vintage. Together they made up over 80% of the fungi detected (Figure 2 and Supplementary Table S4).

### 3.4. Alpha and Beta Diversity Measure

**Figure 3:**
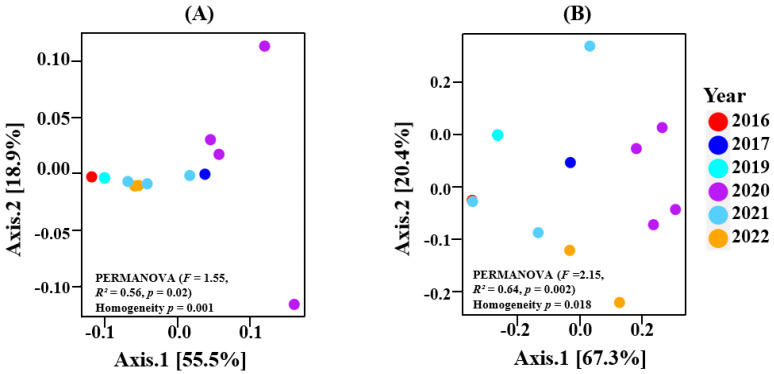
Beta diversity PCoA (Weighted Unifrac) for (**A**) bacterial; (**B**) fungal. Significant PERMANOVA results indicated that the vintage had an impact on the community structure of bacteria and fungi.

The within-sample diversity showed no significant differences for both bacterial and fungal communities across the vintages. Nevertheless, the higher richness and diversity associated with the 2020 vintage is highlighted in both bacterial and fungal plots. This is shown in Supplementary File S2. For beta diversity, our results indicated that the bacterial (PERMANOVA—F = 1.55, R^2^ = 0.56, *p* = 0.02) and fungal (PERMANOVA—F = 2.15, R^2^ = 0.64, *p* = 0.002) composition differed across all vintages (Figure 3).

### 3.5. Influence of Climate on Microbial Community Composition in Oamaru-Brewed Beers

**Figure 4:**
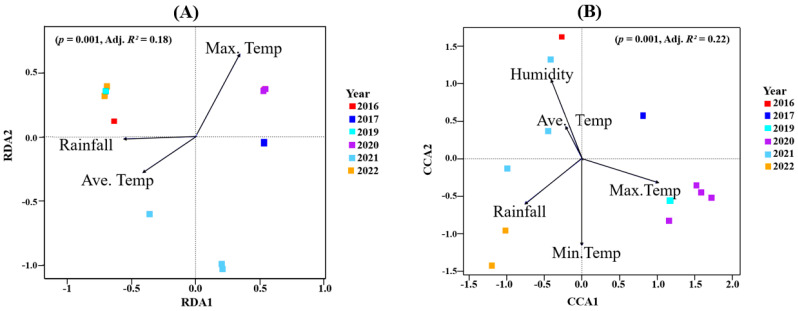
(**A**) Redundancy analyses (RDAs) for bacterial communities. (**B**) Canonical correspondence analysis (CCA) for fungal microbiome. The arrows were the climatic data used to constrain the axes. The angles and lengths of the arrows show the correlations between the first two axes and the climatic variables. Site scores, site constraints and biplot scores are shown in Supplementary Table S6.

The model (*p* = 0.001; Adj. *R*^2^ = 0.18) that best described the bacteria data had the average temperature, maximum temperature, and rainfall as constraining variables (Figure 4A, redundancy analysis, RDA). Of the total inertia, 40.38% was explained by the RDA constrained axes. Maximum temperature had the highest impact on the first and second RDA axis. The site constraints indicated that all four samples (barrels) of the 2020 vintage as well as the 2017 vintage were positively correlated with the first RDA axis, thus suggesting that maximum temperature might explain the bacterial community composition detected in those vintages (Figure 4A). Similarly, samples from the 2016, 2019, 2021 and the 2022 vintage all strongly negatively correlated with the first RDA axis. This indicated that average temperature and rainfall influenced their bacterial composition. A further positive correlation between rainfall and average temperature was observed with both variables negatively correlated with maximum temperature. These results were confirmed using ANOVA (by terms): average temperature (*F* = 1.57, *p* = 0.021), maximum temperature (*F* = 1.67, *p* = 0.033) and rainfall (*F* = 2.17, *p* = 0.002) (Supplementary Table S5A).

For the fungal data, canonical correspondence analysis (CCA) was used to uncover climatic variables that best explain the variance observed in the data (Figure 4B). Here, the model (*p* = 0.001; Adj. *R*^2^ = 0.22) where five climatic variables were included explained a better proportion of the observed variance. The constrained axes described 57.09% of the total inertia detected. The first CCA axis was positively correlated with maximum temperature, while it was negatively correlated with all other variables. The second CCA, on the other hand, was positively correlated with humidity and average temperature and negatively correlated with minimum temperature, max. temperature and rainfall. Here, humidity and average temperature were positively correlated, while both were negatively correlated with maximum temperature. At approximately right angles to each other, rainfall and humidity were uncorrelated. Upon observing the site constraints, we inferred that maximum temperature had the highest impact on the fungal community composition from the 2017, 2019 and 2020 vintages. The 2016 and 2021 vintages were influenced by average temperature and humidity. The minimum temperature and rainfall appeared to drive the 2022 fungal community (Figure 4B). Supplementary Table S5B shows the statistical significance of all explanatory variables.

### 3.6. Organic Acid Concentrations and Correlations with Microbial Data

Statistical analysis of organic acid data revealed that the concentrations of acetic acid and citric acid were consistent across all vintages. However, statistically different concentrations were found in L-malic acid, succinic acid, and L-lactic acid. The highest concentration of L-malic acid was detected in the 2021 vintage, while the lowest was found in the 2022 vintage. The 2017 vintage had high concentrations of succinic acid, and again, the smallest amounts of this acid were reported in the 2022 vintage. These are shown in Table 3.

**Figure 5:**
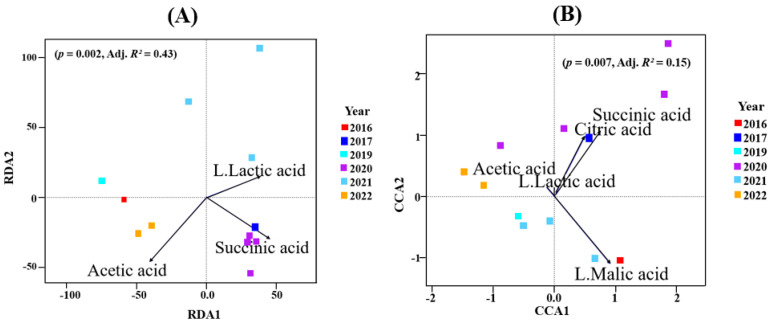
(**A**) Redundancy analyses (RDA) for bacterial communities. (**B**) Canonical correspondence analysis (CCA) for fungal microbiome. The arrows were the organic acid concentrations used to constrain the axes. The angles and lengths of the arrows shows the correlations between the first two axes and the organic acids. Site scores, site constraints and biplot scores are shown in Supplementary Table S8.

When organic acid concentrations were correlated with microbial data using RDA, the best model (*p* = 0.002; Adj. *R*^2^ = 0.43) for the bacterial data had succinic, lactic and acetic acid as constraining variables (Figure 5A; Supplementary Table S8). The constrained axes explained 58.6% of the total inertia. The first RDA axis correlated with succinic (F = 5; *p* = 0.007) and lactic (F = 4.49; *p* = 0.008) acid. This may indicate that the bacterial community in those vintages (2017, 2020, 2021) may be responsible for this (Figure 5A and Supplementary Table S7A).

Similarly, using organic acids as constraining variables via CCA for the fungal data accounted for 53% of the total inertia. Here, the model (*p* = 0.007; Adj. *R*^2^ = 0.15) that best described the data used citric acid, malic acid, succinic acid, lactic acid and acetic acid. Figure 5B shows that the first CCA correlated positively with citric, malic and succinic acid. All these were reaffirmed by ANOVA (by terms) (Supplementary Table S7B).

### 3.7. Analysis of ASVs That Were Differentially Abundant in All Vintages

The list containing the bacterial ASVs that were differentially expressed are shown in Supplementary Table S9. None were reported between vintage 2016 and 2017, vintage 2016 and 2019, vintage 2016 and 2020 and vintage 2016 and 2021. At the genus level, the most differences were identified between vintage 2021 and vintage 2022 where 12 ASVs were noted. Some examples include *Klebsiella* (log 2-fold change; −26.9; *p.adj*; 1.82 × 10^−7^), *Komagataeibacter* (log 2-fold change; 24.7; *p.adj*; 2.36 × 10^−7^), and *Pediococcus* (log 2-fold change; 14.8; *p.adj*; 1 × 10^−3^). In some cases, it was observed that some ASVs identified at a certain taxonomic level (e.g., *Pediococcus*) had both a positive and a negative log 2-fold change. An ASV identified to the family level (Solirubrobacteraceae) was differentially abundant and specifically associated with the 2020 vintage.

For the fungal data, only the vintage 2016 and 2017 showed no differentially abundant ASV. This implies that more differential ASVs were associated with the fungal data as compared with the bacterial data. Unlike the bacterial data, the most differential fungal ASVs (16) were noted between vintage 2020 and vintage 2021. These include *A. pullulans* and *H. valbyensis* with higher differential abundance in the 2020 vintage than those observed in the 2021 vintage. ASVs that were differentially abundant in the 2021 vintage were identified as *Metschnikowia* and *Cladosporium*. All these are shown in Supplementary Table S10.

## 4. Discussion

We assessed the bacterial and fungal diversity in six vintages of a lambic styled beer produced in Oamaru, Otago, New Zealand. Furthermore, we explored the prospect of climatic variables influencing the microbiome of this beer style and compared our results with those of similar studies of Belgian lambic beer [2,16], for which climate data were accessible.

Studies involving the use of metabarcoding to determine the microbiomes of spontaneously fermented beers are, at present, scarce, and to our knowledge, they are otherwise hitherto limited to Belgium [2,16] and Russia [1]. The latter study examined a range of commercially available bottled products without stipulation of production times or locations, making a detailed comparison of their results with those of the Belgian studies or ours limited. Nonetheless, it is relevant to note that the microbial diversity of the Russian products examined was somewhat low, with Lactobacillaceae dominating bacterial presence, and *Brettanomyces* the predominant yeast species found, with exceptions found in three beers where *Saccharomyces* species were dominant. In one of these, *Brettanomyces* represented 45.4% of the yeast population (to 54.1% for *Saccharomyces*), and there was another with *Issantchenkia orientalis* (also known as *Pichia kudriavzevii*) comprising 22.4% of the yeast population. One other beer with *Brettanomyces* comprising 81.5% of the yeast population also had 11.5% *P. kluyveri* present. In summary, over 90% of the bacterial or fungal populations in these beers were represented by one or two taxa only.

Belgium-made lambic beers seem to follow a similar pattern. Bacteria were not detected beyond 13 months, but studies so far identify either *Pediococcus damnosus* [16] or *Acetobacter lambici* [2] as the taxa exclusively identified at this point, whereas at 24 months and 30 months, respectively, the only yeast species detected were *Dekkera (Brettanomyces) bruxellensis* (24 months) or *B. custersianus* (30 months) [2,16].

Our results from end-point examinations of beers produced in Oamaru from 2016 until 2022 display a markedly different pattern of microbial diversity. Only in vintages of 2016 and 2019 do the bacterial populations resemble, in terms of diversity, those outlined above for Belgian and Russian ales with a predominance of *Pediococcus* (Figure 1). However, although the *Pediococcus* species is a notable presence during 2021 and 2022 brewings, it is not exclusive; and for 2017 and especially 2020, it is either absent or marginalized among other bacteria detected. Furthermore, the identity of fungal taxa continually shifts between each year studied (Figure 2); and for 2020 and 2021, where several distinct barrels were investigated, each sample displays a notable microbial population shift.

We have previously inferred a relationship between certain climatic variables and microbial populations in organic wines produced in Waipara [14], which is a region just 300 km from the participating Oamaru brewery. On this basis, we sought to explore the potential influence of such climatic variables on the microbiomes of the beers we examined. Overall, variance in temperature was indicated as the most likely common feature to correlate with the taxonomic variance we observed (Figure 4). However, this is at odds with the temperature data we acquired for Brussels (Table 2), located only approximately 21 km from the Senne valley brewery, where the minimum and maximum temperature ranges are greater than Oamaru. Details of production dates, times and conditions are not provided in the Russian study [1], but as a region, it is certainly far colder [33] than either Belgium or New Zealand, yet their ales display a microbial homogeneity similar to Belgian lambic beers.

What distinguishes New Zealand from both Belgium and Russia is history. The latter two nations have brewed beers since the 13th and 15th centuries, whereas New Zealand’s first record of beer production was by Captain Cook in 1773 with larger production runs established in the 1930s and, more pertinently, with the brewer producing the lambic-style beers examined in this study established only in 2014. We consider it highly likely that during a substantive period of brewing, the indigenous microbes present in Belgian and Russian breweries have become well established and adapted to their environments, in stark contrast to those present in Oamaru, where the brewing conditions are very new by comparison. In this respect, the New Zealand situation is unique, and it is one in which microbial fluctuations, perhaps in part due to climatic variables, and also to a relatively close proximity to the Pacific Ocean, may be better explained.

For Oamaru-produced beers, there was no significant difference between the alpha diversity of all vintages in both bacterial and fungal populations (Supplementary Figure S1), yet beta diversity (PERMANOVA) reported significant differences (Figure 3). Using climatic variables (maximum temperature, minimum temperature, average temperature, rainfall, humidity) and organic acid concentrations (citric acid, L-malic acid, succinic acid, L-lactic acid, acetic acid) as constraining variables associated with the vintages, we uncovered some relationships via RDA and CCA. It appears that bacterial and fungal communities responded differently to these variables. In terms of climate, Figure 4A indicates that maximum temperature was the key factor that influenced the bacterial community associated with the 2020 vintage. The higher bacterial richness and diversity associated with this vintage can be seen in Figure 1D. For fungi, in addition to the 2020 vintage, the fungal community compositions of the 2017 and 2019 vintages were impacted by the maximum temperature (Figure 4B). Similarly, the bacterial composition of the 2020 and the 2016 vintage might have influenced the concentrations of succinic acid reported in these vintages (Figure 5A). An identical conclusion can be drawn from the fungal plot (Figure 5B), although it is much more pronounced in the former. Contrasting responses of bacteria and fungi to vintage and site-specific effects have also been reported in wine grapes [34]. Acknowledging the unique position of New Zealand in the history of brewing, we believe ours is the first study to report these observations: further studies of the impact of these variables in organic beer fermentation would be helpful to confirm this association.

Overall, besides the 2020 vintage, *Pediococcus* is a generally significant presence (Figure 1). This is known as one of the lactic acid bacteria (LAB) species commonly seen in a beer environment [6,35,36]. From the outputs of DESeq2, we can infer that different species and/strains of *Pediococcus* might dominate different vintages. However, these could not be resolved beyond the genus level (Supplementary Table S9). Members of the Enterobacteriaceae family have established roles in the beer fermentation process [37]. In our study, these were mainly detected only in the 2021 and 2022 vintages. In other vintages, they were relatively minute (less than 1%–vintage 2019) or absent (2016, 2017 and 2019). Most beer brewers usually acidify their wort manually, and this impacts the growth of members of Enterobacteriaceae [6,38]. Here, this could possibly imply that the brewery employed an acidification approach for earlier vintages, while the latter vintages (2021 and 2022) were not acidified.

The bacterial family Solirubrobacteraceae (log 2-fold change; 22.37; *p.adj*; 2.16 × 10^−8^) was significantly expressed; we believe this is the first time this bacterial family was detected in beer microbiota. These bacteria have previously been isolated from soil microbiomes from tea plantations [39] and wheat cropping systems [40]. Liao et al. [41] suggested that an increase in Solirubrobacteraceae could imply changes in microbial communities from changes in wider ecological conditions. It is interesting to note that 2020 experienced the hottest maximum temperatures and lowest values for rainfall, humidity, wind and gust strength out of all the vintages studied (Table 1). Since maximum temperature as indicated by our RDA plot (Figure 4A) had a significant impact on the bacterial community of the 2020 vintage, this might have been the major driver of this bacterial family. More research will be required to verify this.

For fungi, the filamentous fungi *Penicillium* was conspicuous in all vintages, constituting more than 70% in the 2016 vintage (Figure 2). This has also been identified in other beer studies [35,42]. Specifically, in their study, Piraine et al. [43] found one sample with a high proportion (>80%) of its fungal ASVs linked to *Penicillium*. Bossaert et al. [35] hypothesized that such an observation might be linked to low levels of bitterness and/or alcohol, which provides an environment that is less stringent. In this current study, rainfall and humidity appear to influence the fungal community of the 2016, 2021 and 2022 vintages (Figure 4B). Together these vintages had high proportions of ASVs linked to *Penicillium* (Figure 2). These species are known to thrive with excessive moisture [44]. The correlation between environmental moisture and humidity associated with a given vintage, and the proportion of filamentous fungi such as *Penicillium*, appears logical.

The genera *Brettanomyces, Saccharomyces* and *Hanseniaspora* were detected in all vintages. *Brettanomyces* is known to be abundant in Belgian lambic and gueze beers where it contributes a unique taste [45,46,47,48]. Like wine, the presence of *Saccharomyces* in beer is common [1,2]. They are mainly responsible for converting fermentable carbohydrates into ethanol and carbon dioxide via the Embden–Meyerhof–Parnas pathway [6]. The non-*Saccharomyces* yeasts *Hanseniaspora* is well established in grape and wine fermentation microbiomes [14,49,50]. In beer brewing, the impact of *Hanseniaspora* on the volatile composition of beer have been described [51]. In this study, the 2020 vintage had the highest relative abundance of *Hanseniaspora* (Supplementary Table S4). With maximum temperature having a significant effect on its fungal community composition (Figure 4B), it might be the case that *Hanseniaspora* thrives in vintages associated with higher temperatures. This result is supported by an earlier report where *Hanseniaspora* species were among the first microorganisms to grow when the temperature reaches 30 °C in cocoa and mixed beers fermentation [52,53]. The genus *Kregervanrija* was also detected in one sample of the 2021 vintage and the 2022 vintage. This has previously been isolated from Austrian wines [54], beer, cider and table olives [55]. Amongst other species, De Roos et al. [42] also reported their abundance in the interior surfaces of casks used for lambic beer production. Their detection in these vintages might be due to the retired wine casks/barrels used for beer brewing here.

The organoleptic properties of beer are known to be impacted by organic acids and other compounds such as CO_2_ and ethanol [56,57]. The role of non-*Saccharomyces* yeasts in shaping the mouth feel properties of beer has been discussed [58,59]. L-Lactic acid concentrations varied significantly across the vintages. The highest concentrations occurred in the 2020 and 2021 vintages. Lower amounts were associated with the 2016, 2017 and 2019 vintages. This is in line with Postigo et al. [37] who reported varying concentrations of this acid in experimental beers and suggested that the competition between various yeasts species and those between yeast and lactic acid bacteria (LAB) may be responsible for this. Beers with a sour taste are usually characterized with high lactic acid concentration [60]. This could mean that beers from the 2020 and 2021 vintages were sour compared to the others. The beer from the 2017 vintage will likely be bitter and salty, as it had the highest amounts of succinic acid (Table 3). Bitter flavors and saltiness in beer taste have been attributed to succinic acid [61]. In the brewing process, most breweries aim to minimize succinic acid concentration to improve drinkability [62]. Significant variations were also detected for L-malic acid concentrations. As with citric acid, its level in the starting wort determines its concentration in beer products [63].

No significant difference was observed when the citric acid concentrations in all beers were determined. Although no statistical differences were reported, the variations in amounts reported can be observed. This might be due to the varying amounts of citric acid in the starting wort. Li and Liu [63] have earlier established that this acid is not produced during fermentation and that the concentration in wort primarily determines its amount in beer. The same can be said of acetic acid, where no significant variations were detected in their concentration in all vintages (Table 3).

At this point, we are unsure what this might mean especially from the sensory/organoleptic point of view. Additional studies will be needed to evaluate this.

## 5. Conclusions

In comparison with spontaneously fermented beers produced in Russia and Belgium, the New Zealand equivalents examined in this study display a substantive degree of microbiological flux. The relative stability of beer microbiomes in the other countries for which data are available is likely to be attributed to the lengthy production history and hence adaptation of the indigenous microflora to those local brewing conditions. The production history of the brewery in Oamaru (and New Zealand generally) is chronologically very short, potentially explaining the lack of microbiological coherency between brews and greater susceptibility to climatic variables, as indicated in this study. Additional studies exploring microbial flux in other breweries across New Zealand (and indeed any nation where beers are produced using indigenous microflora) would be valuable to better inform this hypothesis; however, our observations may also be prudent for any new enterprise wishing to embark upon brewing using a spontaneous fermentation approach, where indigenous microflora is not established, and where microbial flux may be influenced by external factors, including those of climatic origin. This could be relevant in generating the consistency of the final product and thus of consequence for the beer industry at large.

## Figures and Tables

**Table 1:** Weather report associated with the northern Otago region. Values are reported as average ± standard deviation (SD).

Vintage	Min. Temp. (°C)	Average Temp. (°C)	Max. Temp. (°C)	Rain (mm)	Humidity (%)	Average Wind (km/h)	Gust (km/h)
**2016**	1.4 ± 3.23	11.5 ± 3.61	24.7 ± 4.80	39.8 ± 23.8	82.9 ± 15.3	5.9 ± 2.05	67.5 ± 9.92
**2017**	1.5 ± 3.26	10.5 ± 3.46	23.8 ± 5.68	57.6 ± 35.3	86.7 ± 9.6	4.2 ± 1.41	58.8 ± 9.95
**2019**	2.1 ± 3.02	11.5 ± 3.4	24.9 ± 5.6	51.0 ± 53.5	76.4 ± 16.3	2.65 ± 2.46	37.31 ± 29.10
**2020**	1.8 ± 3.62	11.1 ± 3.38	25.7 ± 5.17	30.8 ± 20.5	71.9 ± 16.7	0.98 ± 3.4	4.33 ± 14.61
**2021**	1.1 ± 2.76	11.39 ± 3.03	23.2 ± 6.81	42.5 ± 35.1	83.2 ± 14.1	6.28 ± 2.83	56.32 ±19.75
**2022**	2.2 ± 3.34	11.1 ± 3.05	23.8 ± 4.35	62 ± 53.1	75.5 ± 14.5	6.91 ± 1.87	58.95 ± 15.12

**Table 2:** Temperature (°C) minima, maxima, means and standard deviations (SD) for Oamaru and Brussels production regions.

Region	Minima/SD	Mean/SD	Maxima/SD
**Oamaru**	1.68 ± 3.2	12.4 ± 3.98	24.35 ± 5.4
**Brussels**	−4.25 ± 1.3	11.26 ± 7.25	32.5 ± 0.5

**Table 3:** Organic acids reported in Oamaru-produced beer. Results shown as mean ± standard deviation (SD). Different superscripts in the same column signifies significant differences (Tukey’s HSD test, *p* < 0.05).

Sample ID	Sample	Vintage	Citric Acid (mg/L)	L-Malic Acid (mg/L)	Succinic Acid (mg/L)	L-Lactic Acid (mg/L)	Acetic Acid (mg/L)
1	OBYR16	2016	36.49 ± 1.03 ^a^	805.68 ± 7.54 ^ab^	1518.42 ± 21.33 ^a^	2059.67 ± 1.50 ^b^	2719.22 ± 7.24 ^a^
2	OBYR17	2017	164.29 ± 2.34 ^a^	554.57 ± 5.95 ^c^	7853.93 ± 19.09 ^b^	2378.92 ± 7.85 ^b^	1935.60 ± 24.09 ^a^
3	OBYR19	2019	91.38 ± 0.57 ^a^	632.50 ± 1.51 ^bc^	1355.53 ± 1.03 ^a^	3996.11 ± 48.61 ^b^	2165.75 ± 41.01 ^a^
4	OBYR20A	2020	181.40 ± 1.45 ^a^	668.04 ± 4.54 ^c^	5219.40 ± 11.46 ^ab^	7389.08 ± 4.31 ^a^	2395.66 ± 25.22 ^a^
5	OBYR20B	2020	195.44 ± 0.47 ^a^	646.63 ± 3.72 ^c^	3760.15 ± 9.05 ^ab^	9002.02 ± 21.01 ^a^	540.26 ± 3.20 ^a^
6	OBYR20C	2020	849.73 ± 1.91 ^a^	607.24 ± 1.51 ^c^	5087.91 ± 2.80 ^ab^	7741.88 ± 52.59 ^a^	1308.62 ± 23.91 ^a^
7	OBYR20D	2020	194.73 ± 1.74 ^a^	624.33 ± 2.65 ^c^	3256.08 ± 8.78 ^ab^	8220.19 ± 1.44 ^a^	1040.83 ± 6.00 ^a^
8	OBYR21A	2021	178.76 ± 1.90 ^a^	874.71 ± 2.76 ^a^	1357.05 ± 2.50 ^a^	8517.37 ± 4.36 ^a^	346.51 ± 1.00 ^a^
9	OBYR21B	2021	189.55 ± 0.22 ^a^	909.43 ± 1.09 ^a^	1335.33 ± 2.47 ^a^	8831.55 ± 17.25 ^a^	191.48 ± 6.28 ^a^
10	OBYR21C	2021	163.23 ± 1.51 ^a^	821.00 ± 5.64 ^a^	3051.26 ± 5.24 ^a^	7160.86 ± 6.01 ^a^	208.54 ± 1.61 ^a^
25	NBFM12	2022	142.73 ± 0.26 ^a^	503.93 ± 0.43 ^c^	1092.93 ± 2.47 ^a^	5318.81 ± 7.17 ^ab^	2395.86 ± 18.68 ^a^

## Data Availability

Sequencing data associated with the study can be accessed on the NCBI Sequence Read Archive (SRA) PRJNA1045683. Supplementary figures and tables can be found on Github (https://github.com/Ohwofasa1/Beer-Metabarcoding, accessed 16 October 2024). Brussels weather data purchased from Weatherspark.com can be made available on request.

## Supplementary Materials

The supporting information can be downloaded at https://github.com/Ohwofasa1/Beer-Metabarcoding (accessed on 16 October 2024).
